# The Molecular Mechanism of Craniofacial Cartilage Deformity Induced by High Glucose in Zebrafish

**DOI:** 10.3390/cimb47090687

**Published:** 2025-08-26

**Authors:** Xiaomei Chen, Yong Huang, Xin Yang, Huiqiang Lu, Jian Yang

**Affiliations:** 1 School of Stomatology, Jiangxi Medical College, Nanchang University, Nanchang 330006, China; 363009230001@email.ncu.edu.cn; 2 Jiangxi Provincial Key Laboratory of Oral Diseases, Nanchang 330006, China; 3 Jiangxi Branch of State Key Laboratory for Macromolecule Drugs and Large-Scale Preparation & National Engineering Research Center of Cell Growth Factor Drugs and Protein Biologics, Center for Genetic Development and Regenerative Medicine, Gannan Medical University, Ganzhou 341000, China; yonghuang1996@126.com (Y.H.); 18894452682@163.com (X.Y.)

**Keywords:** gestational diabetes mellitus, cartilage development, cranial neural crest cells, oxidative stress

## Abstract

Gestational diabetes mellitus (GDM), a prevalent metabolic disorder in pregnancy, induces maternal hyperglycemia and elevates fetal malformation risks, particularly in craniofacial development. To investigate the underlying mechanisms, we employed zebrafish as a model organism due to its conserved skeletal development pathways with humans. Zebrafish embryos were exposed to 3.5% and 4% high glucose (HG) from 10–80 h post-fertilization (hpf). Through comprehensive analyses including Alcian blue staining, confocal microscopy, and molecular assays, we demonstrated that HG exposure caused significant developmental abnormalities including growth retardation, craniofacial cartilage malformations, and impaired cranial neural crest cells (CNCCs) migration and proliferation. Mechanistically, HG induced reactive oxygen species (ROS) accumulation and oxidative stress while downregulating critical CNCCs markers (*dlx2* and *tfap2a*). These molecular alterations correlated with histomorphological defects in pharyngeal arch cartilage, particularly in ceratohyal formation. Our findings establish that glucose disrupts craniofacial development through oxidative stress-mediated CNCCs dysfunction, providing novel mechanistic insights into GDM-associated skeletal abnormalities and potential therapeutic targets.

## 1. Introduction

Diabetes mellitus (DM) is a metabolic disorder characterized by chronic hyperglycemia. Based on etiology, it can be classified into four main types: type I diabetes, type II diabetes, gestational diabetes, and other specific types of diabetes [1]. The concept of gestational diabetes mellitus (GDM) was first introduced in 1957 by Elsie Reed Carrington. It refers to abnormal glucose metabolism that develops during the second or third trimester of pregnancy in individuals without pre-existing diabetes [2]. The etiology of GDM is complex, primarily involving pathological processes resulting from physiological changes during pregnancy. These changes include increased glucose demand, enhanced insulin resistance, and relative insulin deficiency. When maternal compensation for these metabolic alterations becomes inadequate, hyperglycemia occurs, ultimately leading to GDM development in some pregnant individuals [3]. Current diagnostic approaches for GDM include fasting blood glucose measurement using glucometers and the oral glucose tolerance test (OGTT) [4]. Epidemiological data indicate that a growing number of pregnant women worldwide are affected by GDM [5].

Recent meta-analyses have demonstrated significant geographical variations in GDM prevalence [6]. The meta-analysis of 32 samples of pregnant women in the US and Canada estimated the prevalence of GDM to be 5.9% [7]. In contrast, a subsequent study employing an inverse square heterogeneity model estimated a 13% pooled prevalence of overall GDM in pregnant women in India [8]. In developed European countries, the estimated prevalence was 5.4%, while data encompassing all European nations reported 10.9% [9,10]. East and Southeast Asia showed a prevalence of 10.1%, with Africa at 13.6% [11,12]. The global prevalence of GDM has shown a progressive upward trend and is projected to continue rising. The rapidly increasing prevalence of GDM poses significant challenges to healthcare systems worldwide. GDM is strongly associated with adverse maternal and neonatal outcomes, presenting substantial risks to both mothers and infants. Current clinical management primarily involves medical nutrition therapy and physical activity for glycemic control. For patients with elevated glucose levels, insulin therapy or other antihyperglycemic medications may be required to achieve optimal glycemic targets and improve pregnancy outcomes [13].

Glucose, derived from maternal dietary carbohydrate metabolism, serves as the primary substrate for intrauterine growth. GDM induces persistent maternal hyperglycemia during pregnancy [14]. Prolonged maternal hyperglycemia significantly elevates the risk of fetal malformations, particularly during early organogenesis, with potential impacts on nearly all organ systems [15]. Research demonstrates that GDM increases the incidence of congenital anomalies, most commonly affecting the cardiovascular and nervous systems, followed by the digestive, musculoskeletal, and renal systems [16,17]. Maternal hyperglycemia during early gestation is strongly associated with fetal malformations. Studies indicate that GDM increases the risk of congenital renal anomalies by 39% compared to normal populations, while the risk of holoprosencephaly rises tenfold in offspring of mothers with GDM [18,19]. Among various malformations, the mechanisms underlying GDM-induced cardiovascular and neurovascular abnormalities have been relatively well elucidated. Using a murine GDM model, Nakano et al. demonstrated that hyperglycemia impairs normal cardiac development by promoting cardiomyocyte proliferation while inhibiting cellular maturation, revealing molecular mechanisms of glucose metabolic dynamics in fetal cardiogenesis [20,21]. While previous studies have also reported GDM-associated skeletal abnormalities, particularly in craniofacial cartilage development, the precise teratogenic mechanisms remain unclear [22,23,24].

The zebrafish (*Danio rerio*), a tropical freshwater fish, has served as a prominent model organism [25]. During larval stages, the minimal pigmentation in zebrafish craniofacial structures enables clear visualization of developmental responses to early drug exposure. The pharyngeal skeleton, which forms the lower craniofacial region, consists of Meckel’s cartilage (MC) and palatoquadrate cartilage (PQ) in the first pharyngeal arch (mandibular arch), interhyal cartilage (IH), hyosymplectic cartilage (HS) and ceratohyal cartilage (CH) in the second pharyngeal arch, and five pairs of ceratobranchial arches (CB1-5) comprising posterior pharyngeal arches 3–7 [26,27]. Morphological alterations in the CH structure serve as reliable indicators of craniofacial cartilage malformations, which can be effectively visualized using Alcian blue and hematoxylin-eosin staining techniques. During craniofacial development, zebrafish pharyngeal arch cartilage originates from cranial neural crest cells (CNCCs). These cells migrate along the anteroposterior axis of developing embryos, following evolutionarily conserved pathways characteristic of vertebrate neural crest cell (NCC) migration [28]. Pharyngeal arch development initiates during segmentation stages, beginning at 10 h post fertilization (hpf) [29]. Midbrain-derived CNCCs commence migration toward chondrogenic regions between 12–18 hpf, with most chondrogenic precursors reaching the pharyngeal arches by 18 hpf and completing migration by 24 hpf. Following migration, these cells undergo proliferation and differentiation, processes that continue until 80 hpf. CNCCs exhibit particular vulnerability to exogenous factors due to their high oxygen consumption and limited antioxidant capacity, making them prone to oxidative damage [30,31]. Consequently, CNCCs are highly susceptible to environmental perturbations, where oxidative stress may disrupt their migration, proliferation, and differentiation during early development, potentially leading to structural organ abnormalities [32,33]. Notably, studies using diabetic pregnancy rodent models have demonstrated that maternal diabetes-induced embryopathy originates from impaired development of neural crest cell derivatives. These findings substantiate that GDM-associated craniofacial anomalies, particularly mandibular dysmorphogenesis, may stem from disrupted CNCCs development [34].

Oxidative stress has been established as a well-recognized pathological condition in various neurological, vascular, and skeletal disorders. This condition primarily arises from excessive production of reactive oxygen species (ROS), which disrupts the redox balance and subsequently leads to cellular damage [35,36]. Consequently, oxidative stress is considered a crucial therapeutic target for numerous systemic diseases. Substantial evidence indicates that certain environmental factors can induce oxidative stress, thereby contributing to cartilage developmental disorders [37]. One study demonstrated that cigarette smoke extract causes varying degrees of craniofacial skeletal defects in zebrafish, including impaired chondrogenesis and bone mineralization [38]. Furthermore, epidemiological data confirm that secondhand smoke exposure during pregnancy adversely affects neonatal skeletal mineralization and disrupts neural crest cell development and migration [38]. These findings collectively suggest that oxidative stress induced by environmental factors plays a pivotal role in disrupting normal craniofacial cartilage development.

This study employs zebrafish as a model organism to investigate the molecular mechanisms by which glucose-induced oxidative stress disrupts cranial neural crest cell migration, proliferation, and differentiation, ultimately leading to craniofacial cartilage malformations.

## 2. Materials and Methods

### 2.1. Chemical and Reagents

All chemicals and reagents were obtained from commercial suppliers. D-(+)-glucose (CAS: A610219) and dimethyl sulfoxide (DMSO; CAS: 67-68-5) were purchased from Sangon Biotech (Shanghai, China). Glucose assay kits (hexokinase method, Cat. No. A154-2-1), total superoxide dismutase (T-SOD, Cat. No. A001-1-2), catalase (CAT, Cat. No. A007-1-1), malondialdehyde (MDA, Cat. No. A003-1-2), and reactive oxygen species (ROS, Cat. No. E004-1-1) assay kits were acquired from Nanjing Jiancheng Bioengineering Institute (Nanjing, China). 5-Fluorouracil (5-FU, Catalog No. HY-90006) was obtained from MedChemExpress (MCE, Shanghai, China).

For histological analysis, Alcian blue 8GX (CAS: 75881-23-1), hematoxylin (CAS: 517-28-2), and eosin (CAS: 15086-94-9) were obtained from Sangon Biotech. Anti-PCNA (CAS: ab29) was sourced from Abcam (Cambridge, UK). All molecular biology reagents for RNA extraction, reverse transcription, and quantitative PCR were procured from TransGen Biotech (Beijing, China).

### 2.2. Zebrafish Maintenance

All zebrafish used in this study were maintained according to standard protocols established by the China Zebrafish Resource Center. Wild-type AB strain and transgenic line *Tg(sox10:GFP)* were acquired from the National Zebrafish Resource Center (Wuhan, China), while the *Tg(col2a1a:Dendra2-NTR)* transgenic line was established in our laboratory in 2022. The fish were housed in a controlled environment with constant water temperature maintained at 28 °C under a 14 h light/10 h dark photoperiod. The recirculating aquaculture system maintained water conductivity at 400–800 μS/cm and pH at approximately 7.0. Zebrafish embryos were collected in dishes post-fertilization for cultivation, with approximately 60 embryos placed per dish. The embryo medium was replaced daily with fresh solution. All experimental procedures were conducted in compliance with guidelines set by the Institutional Animal Care and Use Committee.

### 2.3. High Glucose Exposure Experiment in Zebrafish

Zebrafish embryos were collected in culture medium (0.332 g/L ocean salt, 0.353 g/L NaHCO_3_, pH 7.2) immediately after mating and maintained at 28 °C in a temperature-controlled incubator. At 10 hpf, morphologically normal embryos were selected under a Leica microscope for experimental treatments. A 12% glucose stock solution was prepared by dissolving 12 g D-glucose in 100 mL of 0.0045% 1-phenyl-2-thiourea (PTU) solution and stored at 4 °C. Embryos were exposed to glucose concentrations ranging from 0.5% to 8%. The LC_50_ was calculated using GraphPad Prism 8.0. Based on LC_50_ values and observed craniofacial cartilage malformations, 3.5% and 4% HG concentrations were selected for subsequent experiments. Sample size was determined a priori using G*Power 3.1.9.2 for one-way ANOVA. With an assumed medium effect size (f = 0.25), statistical power of 0.80, and α = 0.05, the analysis indicated a required total sample size of 159 (53 zebrafish embryos per group across 3 experimental conditions). Therefore, embryos were randomly distributed into six-well plates containing either control (8 mL PTU solution) or treatment (3.5% or 4% glucose in PTU) conditions. Each well contained 20 embryos, and three replicate wells were prepared for each concentration to assess mortality rates. To investigate the rescue effects, zebrafish embryos were treated under three experimental conditions from 10 to 80 hpf: PTU, 3.5% HG, 3.5% HG co-administered with 0.25 μM CH223191. The key window for glucose sensitivity was designed using a timed exposure window based on Amitoj Singh’s research [39]. All plates were incubated at 28 °C with daily medium replacement until 80 hpf. Mortality was assessed at 24 h intervals (24, 48, 72 hpf) with three independent experimental replicates.

### 2.4. Quantification of Zebrafish Craniofacial Morphology

At 80 hpf, zebrafish larvae from each high-glucose exposure group were analyzed for growth status using a Leica M205FA stereomicroscope (Nussloch, Germany). Following manual removal of the chorion from unhatched embryos, larvae were transferred to Petri dishes, anesthetized with tricaine, and immobilized in 1 mL of pre-prepared low-melting-point agarose after removing excess liquid. Zebrafish larvae were carefully oriented with fine needles to achieve standardized horizontal alignment of the head, trunk, and tail for imaging. Both bright-field and fluorescence microscopy were performed to document overall morphology and craniofacial cartilage development. Morphological evaluations were conducted by researchers blinded to experimental conditions. Treatment groups were decoded only after completion of all quantitative analyses to prevent observer bias. Morphometric analysis using Image J 1.53 software included measurements of body length, head dimensions, and ceratohyal angle.

### 2.5. Alcian Blue Staining

Embryos at 80 hpf were collected and fixed in 4% paraformaldehyde (PFA) at 4 °C for 36 h. Subsequently, the embryos were treated with 1 mL of bleaching solution (composed of 1.5% H_2_O_2_ and 1% KOH) for 20 min to remove skin pigments. The bleaching solution was then washed off with distilled deionized water (ddH_2_O for 1 h) until complete sedimentation. The embryos were then transferred to 1 mL of Alcian blue staining solution (1 mg Alcian blue dye, 8 mL absolute ethanol, 2 mL glacial acetic acid) and incubated in the dark with shaking for 48 h. Following staining, a graded ethanol dehydration series (95%, 90%, 40%, and 15%) was performed, with each step lasting 2 h. The embryos were then rinsed with ddH_2_O for 2 h. Next, the embryos were treated with 1 mL Proteinase K (1:1000) digestion buffer and placed in a 37 °C incubator to facilitate the digestion of muscle and epidermal tissues. The digestion progress was monitored periodically until the zebrafish cartilage structures became clearly visible under macroscopic observation. The digestion buffer was replaced with 0.5% KOH for two washes (20 min each), followed by sequential treatment with 0.375%, 0.25%, and 0.125% KOH-glycerol solutions under gentle shaking. Finally, the embryos were preserved in pure glycerol for photography.

### 2.6. RNA Extraction and Quantitative PCR Analysis

Thirty zebrafish head tissues at 80 hpf were collected and washed three times with phosphate-buffered saline (PBS) (2 min per wash). Each sample was homogenized in 200 μL of TransZol Up solution using a disposable tissue-grinding pestle on ice for approximately 3 min. An additional 800 μL of TransZol Up solution was added, followed by vigorous vortexing and incubation at room temperature for 5 min. After chloroform extraction, samples were centrifuged at 12,000 rpm for 15 min. The upper aqueous phase (500 μL) was transferred to a fresh microcentrifuge tube and mixed with 500 μL of isopropanol. Following 20 min of incubation at room temperature, RNA was pelleted by centrifugation at 15,000 rpm (4 °C, 15 min). The supernatant was discarded, and the RNA pellet was washed with 75% ethanol. After a final centrifugation (15,000 rpm, 4 °C, 10 min), the supernatant was removed, and the RNA pellet was resuspended in 20 μL of RNase-free water. RNA concentration was determined and stored at −80 °C for long-term preservation.

For cDNA synthesis, RNA samples were reverse transcribed using the TransScript^®^ One-Step gDNA Removal and cDNA Synthesis SuperMix kit. Quantitative PCR (qPCR) was performed using the PerfectStart™ Green qPCR SuperMix detection kit on the Analytik Jena qTOWER3G real-time PCR system (Jena, Germany), with β-actin serving as the internal reference gene. The thermal cycling protocol consisted of an initial denaturation step at 94 °C for 5 s, followed by annealing/extension at 60 °C for 30 s, repeated for 40 cycles. Relative gene expression was calculated using the 2^−ΔΔCT^ method. All primer sequences are provided in Table S1. Each sample was analyzed in triplicate, with data normalized to the control group.

### 2.7. Detection of Oxidative Stress

Zebrafish embryos (*n* = 20 per group) were collected in 1.5 mL centrifuge tubes and washed three times with PBS (2 min per wash). A working solution was prepared by adding 1 μL of 2′,7′-dichlorodihydrofluorescein diacetate (DCFH-DA) fluorescent probe to 1 mL PTU solution, which was then evenly distributed to each tube. Following light protection with aluminum foil wrapping, samples were incubated at 28 °C for 30 min. Embryos were subsequently transferred to six-well plates and subjected to three 10 min washes with working PTU solution before fluorescence imaging. Microscope parameters were standardized by first optimizing exposure settings using control group specimens, then applying identical conditions for all experimental groups during image acquisition through the green fluorescence channel.

Oxidative stress enzyme activities were quantified using fifty zebrafish head tissues per experimental group. Following two washes with 0.9% physiological saline, embryos were homogenized using disposable tissue grinders and centrifuged at 4500 rpm (4 °C, 10 min). Protein concentrations in the supernatants (150 μL aliquots) were determined via Coomassie Brilliant Blue assay using a PerkinElmer microplate reader (Waltham, MA, USA). Protein concentrations were then calculated. Following the operational protocols of the SOD, CAT, and MDA determination kits, samples and reagents were sequentially added. Absorbance readings were converted to enzymatic activities (SOD, CAT) and MDA content using standard calculations.

### 2.8. Proliferating Cell Nuclear Antigen (PCNA) Antibody Staining

Twenty embryos at 80 hpf were collected and fixed overnight at 4 °C using 4% PFA. The samples were dehydrated three times with methanol (30 min each). Subsequently, the embryos were washed three times with 3% PT (3% Triton X-100 in PBS), 30 min per wash. Acetone pre-chilled to −20 °C was added for overnight permeabilization at −20 °C. Excess acetone was removed by washing with 3% PT, followed by tissue digestion with 200 μL proteinase K at room temperature for 20 min to dissociate epidermal structures. The samples were briefly rinsed twice with 3% PT (1 min each) and post-fixed with 4% PFA for 30 min at room temperature. After removing residual fixative with 3% PT, the embryos were blocked in PBTN (3% PT, 4% bovine serum albumin, 0.02% sodium azide) for 4 h at 4 °C with gentle agitation. Primary antibody incubation was performed by adding 200 μL of antibody solution (200 μL PBTN + 0.4 μL PCNA antibody) per tube overnight at 4 °C. Antibodies were removed by washing five times with 3% PT (45 min each). Secondary antibody (500 μL PBTN + 0.25 μL secondary antibody) was applied under light-protected conditions overnight at 4 °C, with subsequent 3% PT washes. Nuclear counterstaining was performed using 200 μL of PBTN containing DAPI (diluted 1:2000; Roche #10236276001, Basel, Switzerland) for 2 h at room temperature. This was followed by three final rinses with 3% PT prior to confocal imaging, which was conducted using a Leica TCS SP8 laser-scanning confocal microscope (Germany). In the 5-FU co-treatment experiment, embryos were treated with 10 μM 5-FU in conjunction with high glucose exposure from 10 to 80 hpf.

### 2.9. Statistical Analysis

Statistical analyses were conducted using GraphPad Prism 8.0, beginning with normality assessment for all experimental groups. Intergroup comparisons were performed using one-way analysis of variance (ANOVA), with all quantitative data presented as mean ± standard error of the mean (SEM). Significance thresholds were established at * *p* < 0.05, ** *p* < 0.01, and *** *p* < 0.001. Morphometric measurements of length and angular parameters were obtained using Image J 1.53 software.

## 3. Results

### 3.1. Early Morphological Analysis of Zebrafish Embryos Following High Glucose Exposure

**Figure 1:**
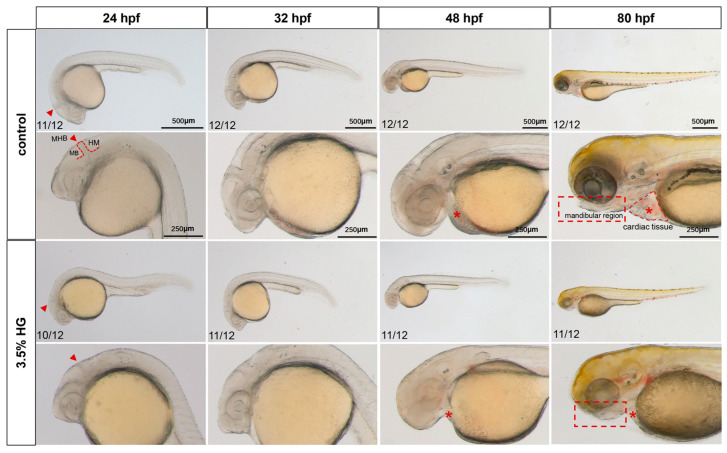
Early morphological analysis of zebrafish exposed to high glucose. Bright-field microscopy revealed systemic phenotypic alterations in zebrafish embryos exposed to high glucose across developmental stages, with cranial magnification to evaluate glucose-induced malformations in early brain regional development (*N* = 12). The midbrain–hindbrain boundary (the developmental boundary separating the midbrain and hindbrain regions) is demarcated by red arrows; cardiac tissue (the developing heart and pericardium) is labeled with asterisks; the mandibular region (the region that will form the jaws) is outlined by a rectangular box. MB: midbrain; MHB: midhindbrain; HB: hindbrain.

Zebrafish embryos exposed to 3.5% HG beginning at 10 hpf exhibited progressive developmental abnormalities across multiple timepoints (24, 32, 48, and 80 hpf). As shown in Figure 1, HG-treated embryos displayed an indistinct midbrain–hindbrain boundary (MHB) by 24 hpf. Comparative analysis at 48 hpf revealed significant developmental defects including cardiac hypoplasia, decreased cranial tissue volume, and yolk sac edema compared with the control group (Figure 1). The most pronounced morphological alterations were observed at 80 hpf, featuring craniofacial retrognathia, reduced head size, and a diminished pericardial area (Figure 1). Interestingly, when glucose was withdrawn at 24 hpf, embryos demonstrated morphological recovery, suggesting the potential for compensatory mechanisms during early development (Figure S3A). To delineate the critical developmental windows of glucose susceptibility, we performed staged exposure experiments in which embryos were treated with high glucose at sequential initiation points (10, 24, 32, and 48 hpf), with all exposures concluding at 80 hpf. This approach revealed a distinct temporal gradient of phenotypic severity, where earlier HG initiation correlated with progressively more pronounced craniofacial malformations (Figure S3B,C). Strikingly, embryos exposed beginning at 48 hpf exhibited no significant morphological differences compared to controls, thereby defining 10–48 hpf as the crucial developmental period of glucose sensitivity (Figure S3B,C). These results demonstrate that embryonic high glucose induces dose-dependent teratogenic effects during critical stages of zebrafish development.

### 3.2. High Glucose Exposure Induces Craniofacial Cartilage Malformation in Zebrafish Embryos

**Figure 2:**
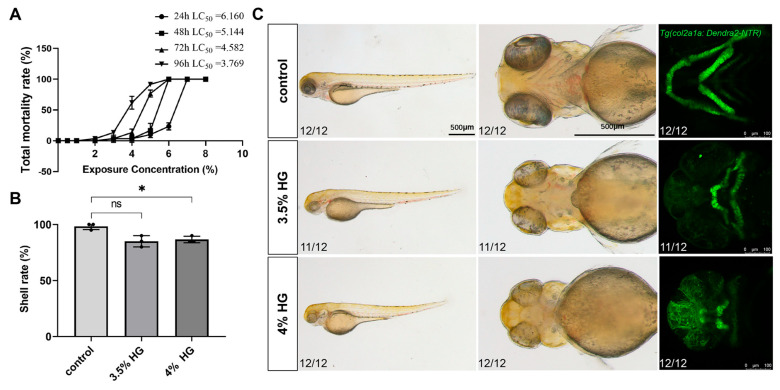
High glucose exposure leads to malformation of craniofacial cartilage development in zebrafish embryos. (**A**) Total mortality rate at 24 hpf, 48 hpf, 72 hpf, and 96 hpf following exposure to different glucose concentrations. (**B**) Hatching rates of zebrafish embryos at 80 hpf after exposure to varying glucose concentrations. (**C**) Representative bright-field and fluorescence images demonstrating the whole-body and pharyngeal arch cartilage phenotypes in zebrafish line *Tg(col2a1a:Dendra2-NTR)* exposed to PTU, 3.5%, and 4% glucose concentrations from 10 hpf to 80 hpf (*N* = 12). Scale bar: 500 μm. Data are presented as mean ± SEM; ns = not significant, * *p* < 0.05.

Following high glucose treatment, the total mortality rates were statistically analyzed at 24 hpf, 48 hpf, 72 hpf, and 96 hpf. The results demonstrated that the LC_50_ values at 24, 48, 72, and 96 hpf were 6.16%, 5.144%, 4.582%, and 3.769%, respectively. Consequently, glucose concentrations of 3.5% and 4% were selected as experimental concentrations (Figure 2A). Chronic exposure from 10–80 hpf induced significant craniofacial abnormalities, including pharyngeal arch cartilage malformations, Meckel’s cartilage defects, and ceratohyal deformities, as confirmed by fluorescent imaging (Figure 2C). Additionally, the hatching rate of zebrafish embryos progressively decreased with increasing glucose concentrations (Figure 2B).

**Figure 3:**
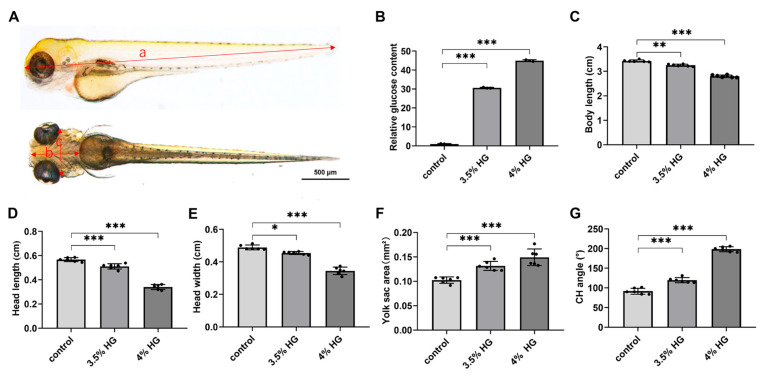
Morphological statistics of craniofacial cartilage of zebrafish exposed to high glucose. (**A**) Standard measurement criteria for zebrafish embryos. a: body length; b: head length; c: head width. (**B**) Relative glucose content at 80 hpf in control and high glucose exposure groups. (**C**–**G**) Morphometric parameters of zebrafish embryos exposed to high glucose at 80 hpf: body length (**C**), head length (**D**), head width (**E**), yolk sac area (**F**), and CH angle (**G**). *N = 12*. Data are presented as mean ± SEM; * *p* < 0.05, ** *p* < 0.01, *** *p* < 0.001.

To further evaluate the morphological changes of cartilage in zebrafish embryos under high-glucose conditions, the transgenic zebrafish line *Tg(col2a1a:Dendra2-NTR)* was employed for assessment. Figure 3A illustrates the measurement methodology for body length, head length, and head width in zebrafish. Morphometric parameters were quantified from standardized views, with body length in lateral view measured as the linear distance from the anterior head margin through the ocular midpoint to the caudal termination. Ventral head length represented the straight-line measurement from the cranial apex to the yolk sac, while head width was defined as the distance between inferior orbital margins. The ceratohyal (CH) angle was determined by angular measurement between bilateral ceratohyal cartilages (Figure 3A). The relative glucose content demonstrated significantly elevated glucose levels in the high-glucose treatment group compared with the control group (Figure 3B). Concurrently, a reduction in body length was observed in zebrafish following high-glucose treatment, along with significant decreases in head length and head width (Figure 3C–E). Additionally, yolk sac area was measured, revealing a progressive increase in response to escalating high glucose concentrations, indicative of potential developmental impairments induced by high glucose (Figure 3F). Morphometric analysis of the pharyngeal arch cartilage demonstrated that the angle between the ceratohyal cartilages exhibited a concentration-dependent increase with higher glucose exposure (Figure 3G). These results demonstrate that high glucose exposure disrupt chondrogenesis and craniofacial morphogenesis during zebrafish embryonic development.

### 3.3. Alcian Blue Staining and HE Staining Showed Craniofacial Cartilage Deformities

**Figure 4:**
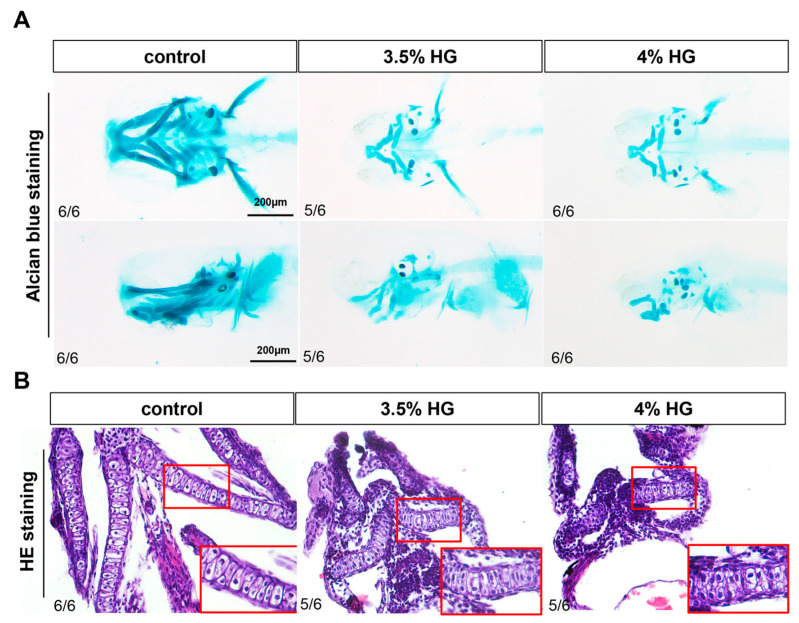
Effects of high glucose exposure on the structure of craniofacial cartilage in zebrafish. (**A**) Alcian blue staining of the control group and glucose exposure group at 80 hpf (*N* = 6). (**B**) HE staining of the control group and high glucose exposure group at 80 hpf (*N* = 6). The inset in the bottom right corner (indicated by the red box) is a magnified portion of pharyngeal arch.

High glucose exposure from 10–80 hpf induced significant craniofacial cartilage malformations in zebrafish, as evidenced by Alcian blue staining. The control group displayed normally organized cartilage architecture, whereas high glucose-treated embryos exhibited progressive deformation of Meckel’s cartilage from oval to triangular morphology, loss of distinct boundaries between palatoquadrate and hyomandibular elements, and abnormal ceratohyal structure (Figure 4A). The branchial arches (1–5) showed pronounced hypoplasia, with 4% glucose exposure causing particularly severe jaw misalignment and cartilage blurring (Figure 4A). Supporting this observation, the results of the relative area of Alcian blue showed that HG exposure decreased the area of the craniofacial cartilage (Figure S2A). HE staining analysis revealed that while control group chondrocytes maintained a characteristic oval morphology with centralized nuclei, glucose-treated specimens contained densely packed, morphologically aberrant cells (Figure 4B). Persistent disorganization of pharyngeal arch cartilage at 5 dpf following continuous 3.5% glucose exposure confirmed that these effects represent structural malformations rather than developmental delays, demonstrating glucose’s teratogenic impact on craniofacial morphogenesis (Figure S1).

### 3.4. High Glucose Exposure Impairs the Proliferation and Differentiation of Neural Crest Cells in Zebrafish

**Figure 5:**
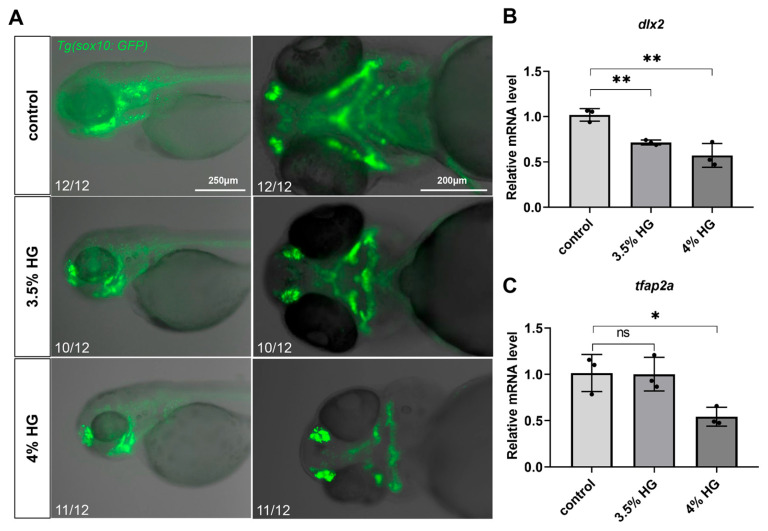
High glucose exposure affects the proliferation and differentiation of zebrafish cranial neural crest cells. (**A**) The distribution of cranial neural crest cells in the pharyngeal arch cartilage of zebrafish at 80 hpf was compared between the control group and the high glucose exposure group (*N* = 12). Scale bar: 250 μm (lateral view), 200 μm (ventral view). (**B**,**C**) Relative mRNA expression levels of *dlx2* and *tfap2a* genes in relation to the development of cranial neural crest cells. Data are presented as mean ± SEM; ns = not significant, * *p* < 0.05, ** *p* < 0.01.

**Figure 6:**
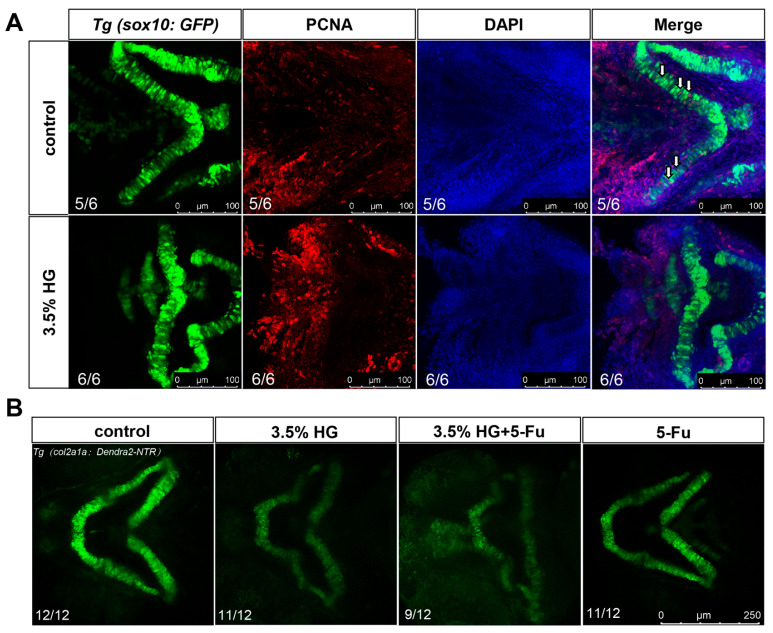
PCNA antibody staining demonstrate reduced proliferation of zebrafish cranial neural crest cells. (**A**) PCNA antibody staining was performed using transgenic zebrafish *Tg(sox10:GFP)*. Neural crest cells are indicated in green, proliferating cells in red, and nuclei in purple (*N* = 6). White arrows indicate proliferating chondrocytes. Scale bar: 100 μm. (**B**) Cartilage development was assessed in transgenic zebrafish *Tg(col2a1a:Dendra-NTR)* under the following experimental conditions: control group, 3.5% high glucose group, 3.5% high glucose group + 5-FU, and 5-FU group (*N* = 12). Scale bar: 250 μm.

Using transgenic zebrafish *Tg(sox10:GFP)*, we observed significant disorganization in the arrangement of NCCs in the high-glucose treatment group, particularly in the CNCCs (Figure 5A). In zebrafish, CNCCs migrate to the pharyngeal arches at early stages and subsequently differentiate into chondrocytes. Although a subset of CNCCs was detected in the ceratohyal (a pharyngeal arch-derived structure) in the high glucose exposure group, the number of ceratohyal chondrocytes was markedly reduced, suggesting potential impairment in the differentiation of CNCCs into chondrocytes (Figure 5A). Compared with the control group, high glucose significantly downregulated the expression of *dlx2* and *tfap2a*, critical regulators of CNCCs proliferation and differentiation (Figure 5B,C). To determine whether high glucose exposure affects CNCCs proliferation, we performed PCNA antibody staining. The results demonstrated a significant reduction in CNCCs proliferation in the 3.5% high glucose group (Figure 6A and Figure S2B). To further investigate the impact of proliferation on chondrocyte development, we co-treated zebrafish with the proliferation inhibitor 5-FU and 3.5% high glucose. The results revealed that proliferation inhibition exacerbated craniofacial cartilage malformations (Figure 6B). Collectively, these findings indicate that high glucose exposure disrupts the normal proliferation and differentiation processes of CNCCs.

### 3.5. High Glucose Exposure Increases Oxidative Stress in Zebrafish Embryos

**Figure 7:**
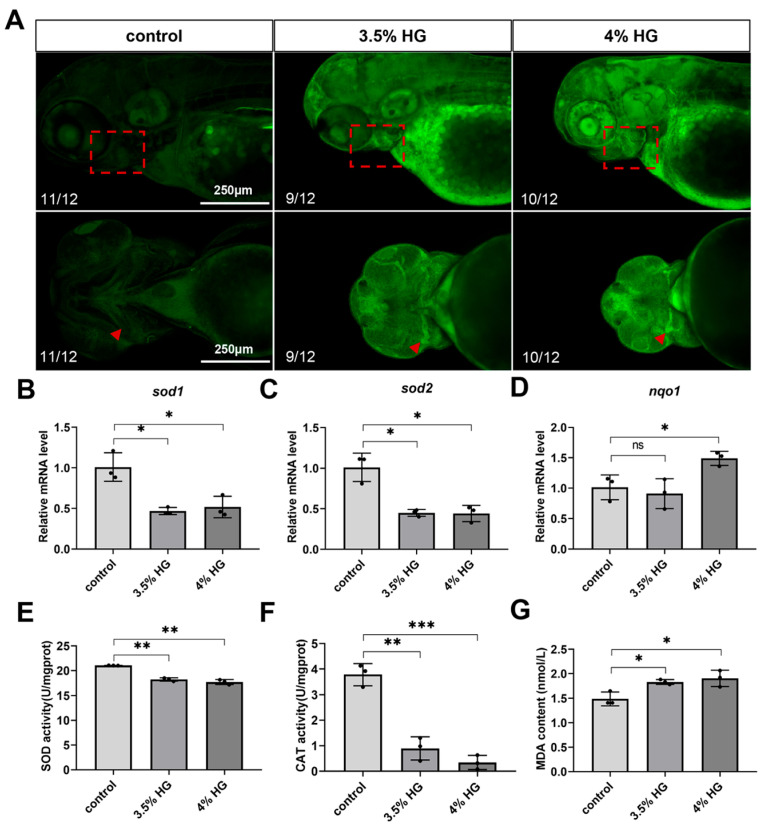
High glucose exposure increases oxidative stress in zebrafish embryos at 80 hpf. (**A**) ROS staining and fluorescence imaging. The boxed area indicates the pharyngeal arch region, with triangular arrows marking ROS accumulation zones in the ceratohyal cartilage (*N* = 12). Scale bar: 250 μm. (**B**–**D**) mRNA relative expression levels of oxidative stress-related genes: *sod1*, *sod2*, and *nqo1*. (**E**) SOD activity. (**F**) CAT activity. (**G**) MDA content. Data are presented as mean ± SEM; ns = not significant, * *p* < 0.05, ** *p* < 0.01, *** *p* < 0.001.

The production of ROS can cause significant oxidative damage to cellular DNA and proteins, ultimately leading to a series of tissue developmental abnormalities. Therefore, we investigated whether zebrafish embryos exposed to high glucose until 80 hpf showed sustained ROS-induced damage. The results revealed significant ROS accumulation in the pharyngeal arch cartilage region of the head (Figure 7A). Subsequently, we measured the relative fluorescence intensity on the ventral side of the pharyngeal arch, revealing that HG exposure increased the accumulation of ROS in the zebrafish pharyngeal arch (Figure S2C). QPCR analysis demonstrated that the high glucose exposure group exhibited significant downregulation of oxidative stress-related genes, *sod1* and *sod2* while upregulating the expression of the stress-responsive gene *nqo1* (Figure 7B–D). Consistent with these findings, the high glucose exposure group showed a marked reduction in the activity of SOD and CAT compared to the control group. Additionally, the levels of lipid peroxidation product MDA exhibited a dose-dependent increase, indicating elevated lipid peroxidation (Figure 7E–G). To investigate the rescue of the pharyngeal arch phenotype, we treated zebrafish embryos with 0.25 μM CH223191. Our results demonstrated that the 3.5% HG + 0.25 μM CH223191 group exhibited a significant rescue of the pharyngeal arch defects (Figure S4A,B). In summary, high glucose exposure induces significant ROS accumulation in the pharyngeal arch cartilage of zebrafish embryos, subsequently triggering an oxidative stress response.

### 3.6. High Glucose Exposure Impairs Neural Crest Cell Migration and Significantly Increases Oxidative Stress at the Early Stage of 24 hpf

**Figure 8:**
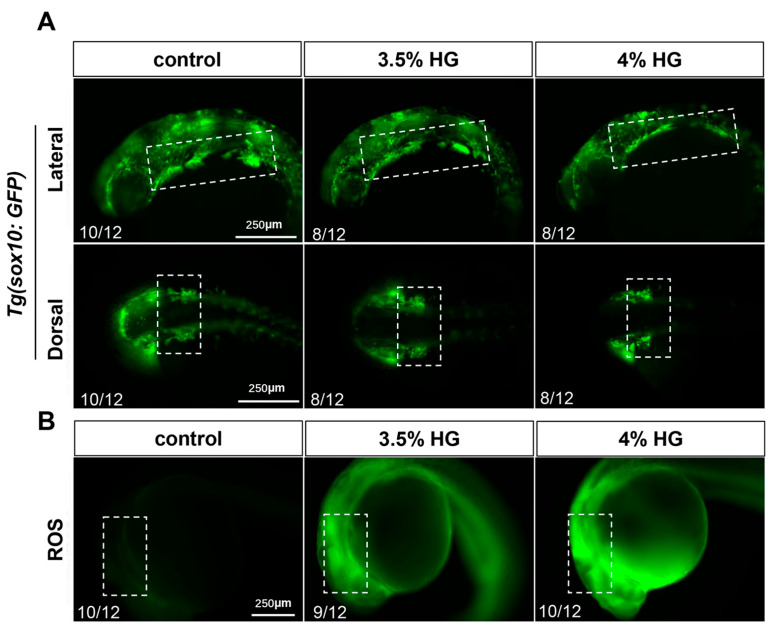
High glucose exposure reduces the migration of neural crest cells and significantly increases oxidative stress at 24 hpf. (**A**) Fluorescence images of *Tg(sox10:GFP)* embryos exposed to 3.5% and 4% high glucose at 24 hpf (*N = 12*). Scale bar: 250 μm. (**B**) ROS staining fluorescence images at 24 hpf. The boxed areas indicate the craniofacial pharyngeal arch cartilage regions (*N = 12*).

Neural crest cells migrate to the pharyngeal arches between 14–24 hpf. To investigate the effects of high glucose on neural crest cell migration, *Tg(sox10:GFP)* transgenic zebrafish embryos were exposed to high glucose from 10 hpf to 24 hpf. The results demonstrated that high glucose exposure led to irregular fluorescence distribution in the craniofacial pharyngeal arches, with a marked reduction in neural crest cells within the sixth and seventh pharyngeal arches, indicating a significant impairment of CNCCs migration (Figure 8A). Concurrently, ROS levels were measured using the DCFH-DA probe in 24 hpf zebrafish embryos exposed to high glucose. The results revealed substantial ROS accumulation in the pharyngeal arch cartilage region, suggesting a pronounced elevation in oxidative stress during this developmental stage (Figure 8B). These findings collectively indicate that high glucose exposure significantly disrupts neural crest cell migration, and this alteration may be associated with high glucose-induced oxidative stress.

## 4. Discussion

DM is recognized as a chronic disorder characterized by hyperglycemia resulting from insulin resistance or subsequent insulin deficiency due to pancreatic β-cell dysfunction [40]. GDM, defined as glucose intolerance with onset or first recognition during pregnancy, has become an increasingly significant public health issue that heightens long-term health risks for both mothers and their offspring [41]. However, mechanistic investigations of GDM-induced developmental alterations in human studies are significantly confounded by numerous uncontrollable variables, including both intrinsic (e.g., genetic predisposition) and extrinsic factors (e.g., dietary patterns and socioeconomic status), which may collectively compromise the intrauterine environment and elevate congenital malformation risks [42]. Therefore, it is imperative to establish an appropriate animal model for mechanistic studies of GDM-associated congenital malformations.

The zebrafish has emerged as an invaluable model organism for biomedical research, possessing 25 chromosomes with extensive gene orthology to human disease-related genes [43,44]. Notably, zebrafish have been widely employed across multiple research domains, including developmental biology, environmental toxicology, oncological studies, human disease modeling, and pharmacological screening. Particularly in skeletal research, zebrafish demonstrate evolutionarily conserved osteoblasts, osteoclasts, and chondrocytes comparable to mammalian systems [45]. Their skeletal tissues can be dynamically visualized through various staining techniques (Alcian blue, calcein, alizarin red) to monitor bone formation and mineralization processes [33,46]. In our study, glucose was employed as an exogenous agent to simulate a hyperglycemic fetal microenvironment in zebrafish embryos. Our findings demonstrate that glucose exposure significantly disrupts the normal development of craniofacial pharyngeal arch cartilage.

Craniofacial malformations represent one of the most common congenital anomalies globally, comprising more than one-third of all birth defects [47,48]. Importantly, numerous critical genes implicated in craniofacial development exhibit remarkable conservation between zebrafish and humans [49,50]. In zebrafish, craniofacial cartilage is primarily formed by CNCCs, which rapidly migrate and subsequent differentiate into craniofacial structures [51,52]. The development of pharyngeal arch cartilage in zebrafish is inseparable from the normal migration of neural crest cells [53,54]. *Dlx2*, a mammalian homolog of the distal-less homeobox gene family, exhibits predominant expression in the mandibular process [55]. Genetic alterations in *dlx2* impair CNCCs migration, proliferation, and differentiation, consequently disrupting normal skeletogenesis [56,57]. Similarly, *tfap2a* demonstrates stage-specific expression throughout NCC development and plays pivotal roles in NCC specification, particularly in regulating CNCCs migration, proliferation, and survival during vertebrate craniofacial morphogenesis [58]. Our investigation revealed significant transcriptional downregulation of both *dlx2* and *tfap2a*, indicating their potential involvement in mediating abnormal NCC proliferation and differentiation patterns in zebrafish embryos. To specifically examine high glucose’s impact on early CNCCs migration, we employed *Tg(sox10:GFP)* transgenic zebrafish at 24 hpf. Additionally, PCNA antibody staining was also conducted on zebrafish pharyngeal arches at 80 hpf. The results indicate that high glucose exposure may cause oxidative stress in the body, induce abnormal migration, proliferation, and differentiation of cranial neural crest cells, and subsequently lead to abnormal cartilage development.

Glucose, derived from maternal dietary carbohydrate metabolism, is the primary substrate for fetal growth and development in utero [59]. Previous animal studies have shown that embryonic exposure to glucose can induce cellular damage, leading to significant morphological abnormalities, potentially mediated by hyperglycemia-induced oxidative stress [60]. In rodent models of GDM, disturbances in oxidative stress, hexosamine and endoplasmic reticulum stress pathways, and autophagy and apoptosis processes have been observed in association with poor glycemic control during pregnancy [61,62]. Extending these findings to humans, clinical case–control studies have shown that women with GDM exhibit elevated levels of oxidative stress, which results from both increased free radical production and compromised antioxidant defenses [63,64]. Emerging evidence further supports the notion that ROS-mediated oxidative stress is a key mechanistic driver of cardiovascular malformations induced by maternal hyperglycemia [65,66]. Collectively, these studies encompassing both animal models and clinical observations emphasize the pivotal role of oxidative stress in mediating the adverse developmental outcomes linked to maternal hyperglycemia.

Concurrently, oxidative stress may profoundly impair developmental processes by simultaneously reducing cellular proliferation, exacerbating apoptosis, and compromising endogenous antioxidant defense mechanisms. Furthermore, this pathological state promotes mitochondrial dysfunction, facilitates lipid peroxide accumulation, and induces DNA damage in embryonic tissues. These interconnected pathological cascades collectively disrupt normal embryogenesis, culminating in neonatal developmental abnormalities [67]. Given the high sensitivity of CNCCs to environmental perturbations, these cells are particularly vulnerable to oxidative damage, which may ultimately result in craniofacial malformations [31,68,69]. Therefore, we measured ROS levels at both 24 and 80 hpf. Previous studies have shown that aryl hydrocarbon receptors (AHRs) are key mediators of organic compound-induced oxidative stress, subsequently leading to DNA damage and apoptosis [70]. In light of these findings, we employed the AHR inhibitor CH223191 and observed a significant reduction in HG-induced craniofacial cartilage malformation in zebrafish embryos. Based on these results, we propose that the craniofacial cartilage malformation induced by HG is likely caused by a mechanism involving oxidative stress. Although the zebrafish embryo model provides distinct advantages for this investigation, several important differences from human fetal development should be acknowledged. Unlike mammalian fetuses that depend on maternal glucose supply, zebrafish embryos initially utilize yolk-derived nutrients [71]. Additionally, species-specific differences in antioxidant defense mechanisms may influence the results. To fully validate these findings, future studies incorporating complementary mammalian models would be valuable.

In this study, zebrafish embryos at 10 hpf were exposed to high glucose concentrations (3.5% and 4%). Morphological and histological analyses were performed to evaluate phenotypic alterations in craniofacial pharyngeal arch cartilage during early development under hyperglycemic conditions. Moreover, we systematically elucidated the inhibitory effects of high glucose on cranial neural crest cell migration, proliferation, and differentiation in zebrafish. Reactive oxygen species levels were also assessed in glucose-treated embryos. This study provides mechanistic evidence linking high glucose exposure to developmental toxicity in craniofacial chondrogenesis.

## 5. Conclusions

This study demonstrates that high glucose exposure (10–80 hpf) induces developmental toxicity in zebrafish embryos, manifested through increased mortality, growth retardation (reduced body length), and multiple morphological abnormalities including pericardial edema, yolk sac edema, and craniofacial cartilage malformations. Mechanistically, glucose-induced oxidative stress disrupts neural crest cell migration, proliferation, and differentiation, resulting in pharyngeal arch defects. These findings establish oxidative stress-mediated NCC dysfunction as a principal mechanism underlying glucose-induced craniofacial developmental toxicity.

## Data Availability

Data are contained within this article and Supplementary Materials.

## Supplementary Materials

The following supporting information can be downloaded at: https://www.mdpi.com/article/10.3390/cimb47090687/s1.
