# Supplementary material for: The Molecular Mechanism of Craniofacial Cartilage Deformity Induced by High Glucose in Zebrafish
Source: Curr Issues Mol Biol. 2025 Aug 26;47(9):687. doi: 10.3390/cimb47090687 (PMC12468894; doi:10.3390/cimb47090687)
Supplement: Supplementary file 1 [file cimb-47-00687-s001.zip › cimb-3827527-supplementary.pdf]

The Molecular Mechanism of Craniofacial Cartilage Deformity Induced by High Glucose in Zebrafish

Supplementary material

1. Supplementary figures and legends:

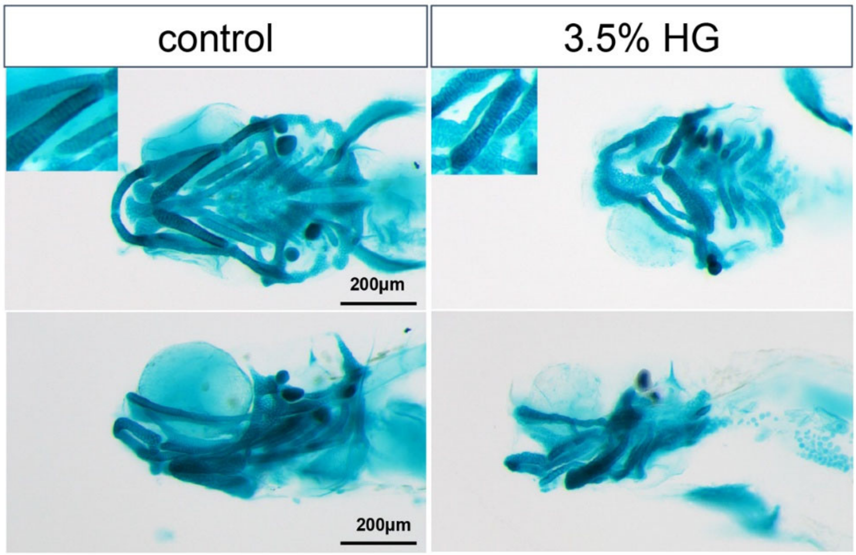

Supplementary Figure S1. Alcian blue staining of pharyngeal arch cartilage in control and 3.5% HG-treated zebrafish embryos from 10 hpf to 120 hpf. Alcian blue staining revealed that the pharyngeal arch cartilage structures in the 3.5% HG group remained disorganized. Scale bar: 200 μm.

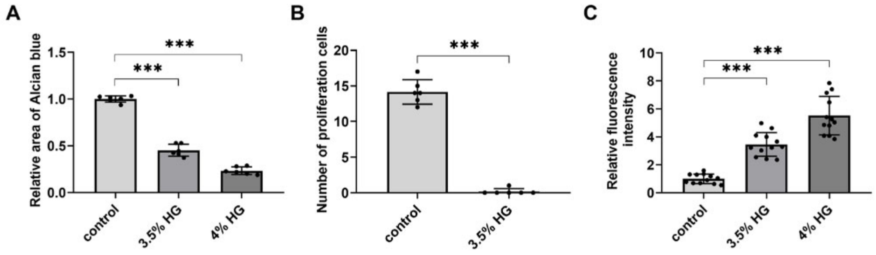

Supplementary Figure S2. Quantitative analysis of craniofacial cartilage morphology and cell proliferation under HG conditions. (A) Relative area of Alcian blue-stained craniofacial cartilage in control and HG-treated embryos at 80 hpf (N=6). (B) Quantification of proliferating cells (PCNA-positive) in the pharyngeal arch cartilage (N=6). (C) Relative fluorescence intensity of ROS staining (N=12). Data are presented as mean ± SEM; *p<0.05, **p<0.01, ***p<0.001.

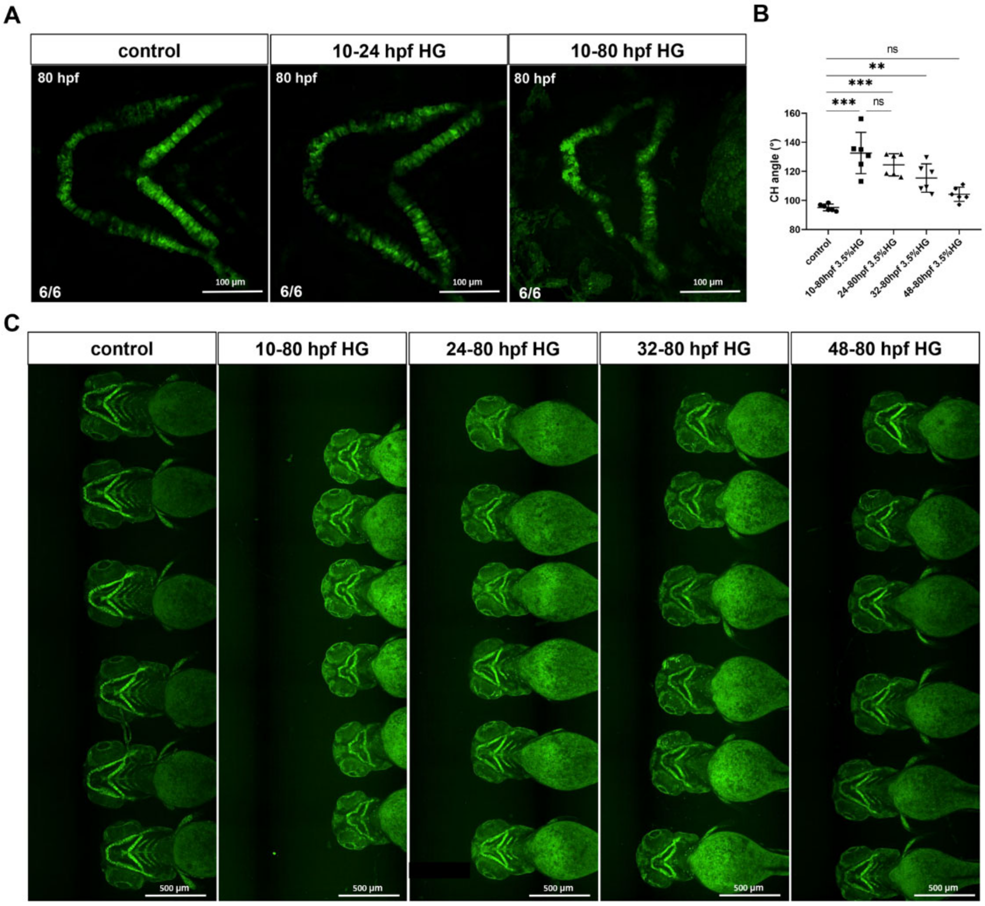

Supplementary Figure S3. Temporal effects of HG exposure on craniofacial development in *Tg(col2a1a:Dendra2-NTR)* zebrafish embryos. (A) Fluorescence images of pharyngeal arch cartilage phenotypes at 80 hpf under different exposure windows: control group (PTU), 10-24 hpf HG followed by PTU group, and continuous 10-80 hpf HG group (N=6). Scale bar: 100 μm. (B) Quantification of CH angle across staged HG exposures. (C) Fluorescence images of pharyngeal arch cartilage phenotypes at 80 hpf under staged HG exposures (initiated at 10, 24, 32, or 48 hpf; all terminated at 80 hpf). (N=6). Scale bar: 500 μm. Data are presented as mean ± SEM; **p<0.01, ***p<0.001.

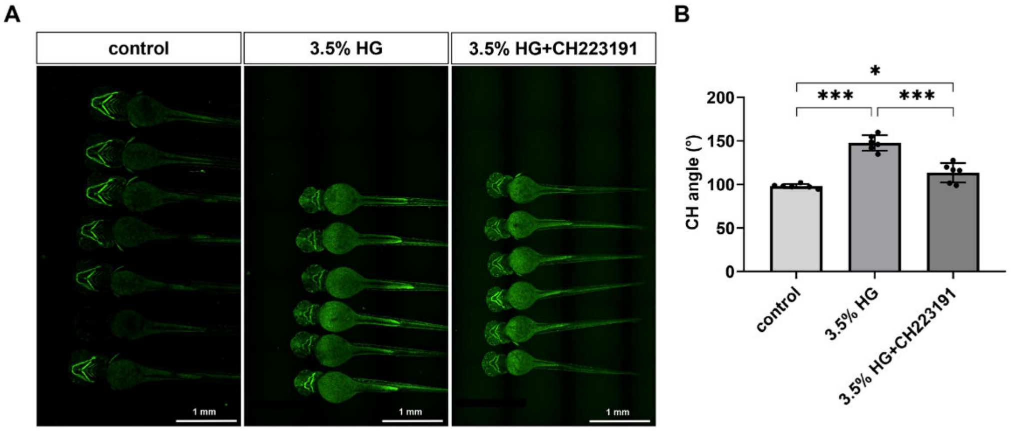

Supplementary Figure S4. Rescue effects of CH223191 on HG-induced craniofacial malformations in *Tg(col2a1a:Dendra2-NTR)* zebrafish. (A) Fluorescent images of chondrocyte-labeled pharyngeal cartilage at 80 hpf following exposure to control (PTU), 3.5% HG, or 3.5% HG with 0.25 μM CH223191 from 10-80 hpf (N=6). Scale bar: 1 mm. (B) Quantification of CH angle. Data are presented as mean ± SEM; $^{**}p<0.01$, $^{***}p<0.001$.

2. Supplementary Table S1: Primer sequences used for qPCR analysis in the article.

gene	**sequences (5'-3') forward**	**sequences (5'-3') reverse**	**Ensemble ID**
dlx2	TCTTCCTGCGAAATGACC	TGTCTGTGAACTGCTGAA	ENSDARG00000079964
tfap2a	ACAAAGTCAGGAGAGCAGCC	TACCACACCTCCAAACAGCC	ENSDARG00000059279
sod1	TGACCGGCACCGTCTATTTC	ATGCAGCCGTTTGTGTTGTC	ENSDARG00000043848
sod2	TGTTGGAGGCCATAAAGCGT	AGTGGGATGAGACCTGTGGT	ENSDARG00000042644
nqo1	TCAAGGCTTCAGCTACTGCG	CTTCTGCGATCAAGCTGAAAGA	ENSDARG00000010250
